# Editorial: Ventricular Mechanics in Congenital Heart Disease

**DOI:** 10.3389/fped.2017.00129

**Published:** 2017-05-31

**Authors:** Giovanni Biglino, Adelaide de Vecchi

**Affiliations:** ^1^School of Clinical Sciences, Bristol Heart Institute, University of Bristol, Bristol, United Kingdom; ^2^Division of Imaging Sciences and Biomedical Engineering, King’s College London, London, United Kingdom

**Keywords:** computational modeling, cardiovascular magnetic resonance imaging, congenital heart disease, ventricular function, patient-specific

**The Editorial on the Research Topic**

**Ventricular Mechanics in Congenital Heart Disease**

American dancer and choreographer Martha Graham (1894–1991) famously said: “Nothing is more revealing than movement.” Indeed, when thinking about the heart and its incessant dance, a huge amount of information can be derived from analyzing the mechanics of the organ, including mechanical impairments in the presence of disease and particularly in the presence of congenital lesions. This Research Topic aims to present recent research findings on ventricular mechanics in congenital heart disease (CHD) patients, alongside with state-of-the-art reviews that expertly summarize current knowledge in this area.

The reviews cover three crucial components. Panesar and Burch elucidate current knowledge of diastolic dysfunction in CHD, an important yet often overlooked component that can lead to heart failure even in patients with normal systolic function (Panesar and Burch). Ghonim et al. review fibrosis and how cardiovascular magnetic resonance (CMR) imaging can aid in its assessment, discussing techniques such as diffusion tensor imaging, tagging, feature tracking, late gadolinium enhancement imaging, and T1 mapping (Ghonim et al.). Finally, Greil et al. look again at the role of medical imaging in this context, but particularly at 3D whole heart CMR imaging, which they indicate as “a cornerstone” in CHD imaging, in the light of its versatile role that includes volumetric analysis in complex geometries, 3D printing, and computational modeling (Greil et al.).

As emerging from this Research Topic, imaging is undeniably a key component when discussing ventricular mechanics, its assessment, and quantification. Original research articles, in fact, demonstrate how different imaging techniques provide us with new insight in CHD patients. Whether assessing patients with tetralogy of Fallot after pulmonary valve replacement (Burkhardt et al.) or the role of isolated tricuspid valve repair in adults with CHD (Marsico et al.), the insight provided by imaging in looking at both right and left ventricle in patients with CHD is undeniable. Specific imaging modalities also play a crucial role in understanding the mechanics of CHDs, from investigating ventricular function in children with dilated cardiomyopathy using late gadolinium enhancement (Muscogiuri et al.) to identify an element of systolic dysfunction in young people with cardiomyopathies and aortic stenosis by means of CMR-derived wave intensity analysis (Ntsinjana et al.). The Research Topic also discusses more methodological considerations with regards to imaging; in particular, Gomez et al. present observations on landmark-based image registration to align CMR and ultrasound images in patients with hypoplastic left heart syndrome and compare ventricular volume measurements obtained with the two modalities (Gomez et al.).

This topic also offers a flavor of applications of computational modeling for the purpose of simulating and better understanding ventricular mechanics. Computational modeling is an increasingly powerful tool that can facilitate a patient-specific approach in assessing different aspects of CHD, from hemodynamics to devices to morphology (1). In this context, three compelling studies well exemplify the breath of applications of computational modeling. A finite element model of the fetal heart, accounting for changes in cardiac myocyte growth rates, is proposed, and its application is shown in both a “normal” scenario and in the presence of hypoplastic left heart syndrome (Dewan et al.). From the point of view of patient-specific modeling, a case study of a 4-month-old patient is presented demonstrating how a multi-domain computational model can recapitulate well the hemodynamics in the Blalock–Taussig shunt and in the pulmonary arteries (Arthurs et al.). Finally, a statistical shape analysis approach is presented as a tool allowing insight into ventricular deformations, presenting models of both patients with repaired aortic stenosis and healthy controls, and putting forward intriguing concepts such as “shape biomarkers” and “motion biomarkers” (Biffi et al.).

What emerges from these studies is certainly the multifaceted nature of analyzing ventricular motion and ventricular remodeling in patients which CHD. While this conclusion is not surprising in itself, these studies and reviews provide an opportunity to reflect on two key aspects of ventricular mechanics in the light of the latest cutting-edge technological progress as follows:

1. Population-based vs. patient-specific: depending on the pathophysiology of each CHD, and the consequent morphological and functional changes in the heart, these two competing philosophical approaches have emerged as effective methodologies to assess ventricular mechanics in the context of the individual and the population, respectively;
2. The role of imaging and modeling: different imaging modalities, and in particular CMR, clearly emerge as an essential tool for generating new knowledge and insight (literally as “looking inside”) into ventricular mechanics in CHD. Similarly, computational modeling has recently reached a stage, where it can be successfully integrated with imaging techniques to enhance diagnosis and outcome prediction.

The contributions chosen for this special issue are not just a collection of the latest progress on the key aspects mentioned above. The purpose of this volume is to present new research that can shed light on these crucial points with the aim to link methodological approaches to clinical outcomes and, ultimately, patient benefit. Placing these recent advances into the end-user context is a key last step that will enable a true and purposeful translation of science: all these original research contributions strive to achieve this goal by proposing methods to test new biomarkers of disease progression. Movement, or the absence of, can be extremely revealing, but to what extent novel analyses will allow not only quantification of movement but also possible clinically meaningful predictions in the light of movement abnormalities? The simultaneous development of advanced imaging techniques, methodologies for image processing, and computational modeling, as well as the relentless progress made by the research community world-wide to integrate these tools both among each other and within clinical practice, can hold the key to this fundamental question. Ultimately, CHDs encompass a wide range of anatomical and functional maladaptive traits, and as such there is no single recipe for positive outcomes in the clinic. Rather, the ability to understand and interpret the pathological changes from a mechanistic perspective is pivotal to select the most effective approach in the context of each disease.

Future directions include continued efforts for large-scale validation of computational models, as well as the development of user-friendly platforms to render the application of patient-specific simulations not only feasible but also accessible and efficient in the clinical domain. The progress in imaging acquisition techniques and processing presented in this Research Topic also holds significant potential for developing disease classifiers from image feature quantification in conjunction with machine learning algorithms. Another exciting area of future development outside the field of imaging and modeling would in fact be to explore the possibility of developing risk scoring methodologies, such as algorithms trained using electronic health records (2). The latter is beginning to show promise in other medical applications.

While some of the studies presented in this Research Topic are based on too small sample sizes to lead directly to clinical applications, the subtlety of the processes analyzed, together with the knowledge that some of the validated indices routinely used in adults are not necessarily informative in children (3), should at the very least lead to an open-minded approach toward exploring novel indices and measures to unravel this problem and improve the prognosis of these patients.

## Author Contributions

GB and AdV have contributed equally to this work.

## Conflict of Interest Statement

The authors declare that the research was conducted in the absence of any commercial or financial relationships that could be construed as a potential conflict of interest.
